# Extra-Intestinal Fluoroquinolone-Resistant Escherichia coli Strains Isolated from Meat

**DOI:** 10.1155/2018/8714975

**Published:** 2018-11-18

**Authors:** Giorgia Caruso, Anna Giammanco, Cinzia Cardamone, Giuseppa Oliveri, Chiara Mascarella, Giuseppina Capra, Teresa Fasciana

**Affiliations:** ^1^Department of Sciences for Health Promotion and Mother & Child Care, University of Palermo, Italy; ^2^Institute for Experimental Veterinary Medicine of Sicily, Palermo, Italy

## Abstract

Extra-intestinal* E. coli* are emerging as a global threat due to their diffusion as opportunistic pathogens and, above all, to their wide set of antibiotic resistance determinants. There are still many gaps in our knowledge of their origin and spread pathways, although food animals have been adjudicated vehicles for passing mult-drug resistant bacteria to humans. This study analyzed 46 samples of meat purchased from retail stores in Palermo in order to obtain quinolone-resistant* E. coli* isolates. Strains were screened for their phylogenetic groups, ST131-associated single nucleotide polymorphisms (SNPs), and then typed by ERIC-PCR. Their set of virulence factors, namely,* kpsMII, papA, sfaS, focG, iutA, papC, hlyD*, and* afa* genes, were investigated and their fluoroquinolone-resistance determinants evaluated. The data obtained show a dramatically high prevalence of multidrug resistance patterns in the Palermo area, with 28% of the isolates having virulence factor genes typical of ExPEC strains. No B2 group or ST131 strains were detected. Moreover, 20% of our isolates showed positivity to all the plasmid-mediated quinolone resistance (PMQR) determinants, showing a potential to transfer these genes among other bacteria. Therefore, these data underline the possibility that food animals and, specifically, poultry in particular may be a significant source of resistant bacterial strains, posing a potential zoonotic risk.

## 1. Introduction

The outburst of the antibiotic resistance phenomenon at global level has occurred due to the excessive and inappropriate use of antimicrobials in various fields, both in human medicine and in veterinary and zootechnical settings, strongly accelerating the development and diffusion of resistant strains. For instance, intensive livestock farming practices that compel farmers to rely more heavily on antibiotics have determined a dramatic increase in the prevalence of antibiotic-resistant bacteria in farm animals and food [1, 2].

Quinolones in particular have long been the main choice of antimicrobial agent for the treatment of various Gram-negative infections, both in human and in veterinary medicine, ostensibly increasing the rate of resistant isolates all over the world [3]. Furthermore, the World Health Organization (WHO) has signalled quinolones to be critically important antibiotics, thus recommending a more prudent use of them [4]. In fact, since the discovery of the first plasmid-mediated quinolone resistance (PMQR) gene in 1998 [5], many other resistance mechanisms have been added [6].

Nontarget, commensal enteric bacteria are also exposed to this wide variety of antimicrobial substances, leading to an increase in resistance genes and, potentially, their horizontal transfer. Hence these bacteria may function as a reservoir of resistance though largely ignored [7].

According to the EFSA and ECDC report [8],* E. coli* is an excellent indicator of resistance level among Enterobacteria in breeding animals, as it is widespread in farm environments.

Furthermore, there is increasing evidence that* E. coli* strains may be conveyed through food and, directly or more likely indirectly, they find their way to humans, accounting for a subset of resistant Extra-intestinal Pathogenic* E. coli* (ExPEC) [9–11]. Indeed, ExPEC increasingly represent an emerging category of pathogens that cause illness in immunocompetent subjects both in nosocomial and in community settings. ExPEC are in fact implicated in a wide range of host diseases and are associated with the vast majority of urinary tract infections (UTIs), as well as neonatal meningitis and bacteremia and animal infective syndromes [12].

*E. coli* ST131 is currently the predominant isolate among ExPEC lineages at global level [13] and is considered to be one of the most virulent bacterial clones, particularly linked to fluoroquinolone-resistance (e.g.,* qnrA*,* qnrB*,* qnrS*, and* aac (6')-Ib-cr* genes) and to extended-spectrum *β*-lactamases, such as CTX-M-15 [14]. Moreover, ST131 isolates are commonly reported to harbour a wide variety of virulence-associated genes, including a greater ability to produce biofilms compared to non-ST131 isolates [15]. Due to these features, ST131 strains are considered to be truly pathogenic [13]. The aim of this study, therefore, was to assess the prevalence of multidrug resistant* E. coli* with ExPEC-associated traits in food that could pose a risk for consumers.

## 2. Materials and Methods

### 2.1. Strain Selection

Between January and March 2017, 46 samples were analyzed at the Experimental Zooprophylactic Institute of Sicily “A. Mirri.” All the samples, in individually sealed packages, were purchased from different supermarkets in Palermo. They consisted of 23 poultry, 13 beef and 10 pork samples. According to the labels, all the samples came from intensive farms based in Sicily.

The samples were immediately sent to the laboratory on ice and subsequently processed in asepsis.

10 g of each sample was added to 90 ml of saline peptone solution (SSP). After homogenization by Stomacher and incubation for one hour at room temperature, samples were plated into Tryptone Bile X-Glucuronide (TBX) Agar. Colonies developed 18 to 24 hours after incubation at 44°C. They were tested by disk diffusion for resistance to fluoroquinolones, in particular to ciprofloxacin (CIP, 5 *μ*g), norfloxacin (NOR, 10 *μ*g), and levofloxacin (LVX, 5 *μ*g) (Oxoid). A resistant colony was selected from each sample and then identified by the API E (BioMérieux) system.

A single colony was suspended in 200 *μ*l sterile bidistilled water. DNA extraction was then performed by High Pure PCR Template Preparation Kit (Roche), according to the manufacturer's instructions.

### 2.2. Phylogenetic Grouping

DNA extracts were analysed with multiplex PCR to ascertain their phylogenetic groups, as described by Clermont et al. [16]. This method is based on the presence/absence of three genes:* chuA*, which encodes a protein transporting the eme group,* yjaA*, with an unknown function, and the fragment* TSPE4.C2*, thought to be within a gene encoding a lipase esterase. Previously studied strains from our laboratory were used as positive controls [17].

### 2.3. Virulence Factors

Two multiplex PCRs were assayed to investigate the presence of eight virulence factors (VFs) in the* E. coli* isolates, as described by Johnson et al. [18]. The first multiplex PCR screened for the presence of* kpsMII *(group II capsule),* papA *(pilus-associated protein A),* sfaS *(S-fimbrial adhesine), and* focG* (F1C fimbriae protein) genes; the second one searched for* hlyD (*haemolysin D*), afa (*afimbrial adhesine),* iutA (*aerobactin siderophore ferric receptor protein), and* papC (*pilus-associated protein C) genes. Positive results for at least two of these VFs are a distinctive sign of ExPEC [18]. Three strains were employed as positive controls:* E. coli* RS218 (*kpsMT II*,* papA*,* papC*,* sfaS*, and* hlyD*),* E. coli* V27 (*kpsMT II*,* papA*,* papC*,* iutA*, and* focG*), and* E. coli* 2H16 (*papC*,* iutA*,* afa*, and* hlyD*) [17].

### 2.4. Genotypic Detection of Plasmid Resistance Genes

Strain genotypes were investigated with relation to the most common plasmid-mediated quinolone resistance genes:* qnrA*,* qnrB*,* qnrS*, and* aac(6')-Ib-cr* [19, 20].

### 2.5. Typing

All the strains were typed in order to screen for ST131-associated single nucleotide polymorphisms (SNPs) in* mdh* and* gyrB*, according to Johnson et al. [21]. Hence a multiplex PCR was carried out, and the presence of the two amplicons relating to the two abovementioned genes qualified the strain as ST131.

They were then subjected to Enterobacterial Repetitive Intergenic Consensus sequence PCR (ERIC-PCR), according to Versalovic et al. [22].

The fingerprints were photographed by the GelDoc (BIO-RAD) system and finally analyzed using the BIO-NUMERICS software (Applied Maths, Kortrijk, Belgium). Comparisons between band patterns were performed with the Dice similarity coefficient. The obtained matrices were combined using the UPGMA algorithm to produce a dendrogram, with a cut-off of 80% similarity.

### 2.6. Antibiotic Testing

Besides fluoroquinolones, all the strains were tested by disk diffusion for susceptibility to other antimicrobials, including amoxicillin–clavulanic acid (AUG, 20–10 *μ*g), cefotaxime (CTX, 30 *μ*g), ceftazidime (CAZ, 30 *μ*g), cefepime (PEP, 30 *μ*g), gentamicin (CN, 10 *μ*g), imipenem (IMI, 10 *μ*g), sulfamethoxazole–trimethoprim (SXT, 25 *μ*g), and tetracycline (TE, 30 *μ*g) (Oxoid). Resistance was determined according to European Committee on Antimicrobial Susceptibility Testing (EUCAST) guidelines (http://www.eucast.org/clinical_breakpoints/). Isolates simultaneously resistant to three or more different drug classes were defined as multidrug resistant (MDR) [23].

## 3. Results

This study analysed 46 samples. Almost all the strains isolated from poultry samples were resistant to fluoroquinolones (91.3%). A considerably lower percentage of the strains isolated from pigs and cattle showed resistance, namely, 20% and 15.3%, respectively. In total, we obtained 25 fluoroquinolone-resistant strains, to be further characterized in the following analyses.

**Figure 1:**
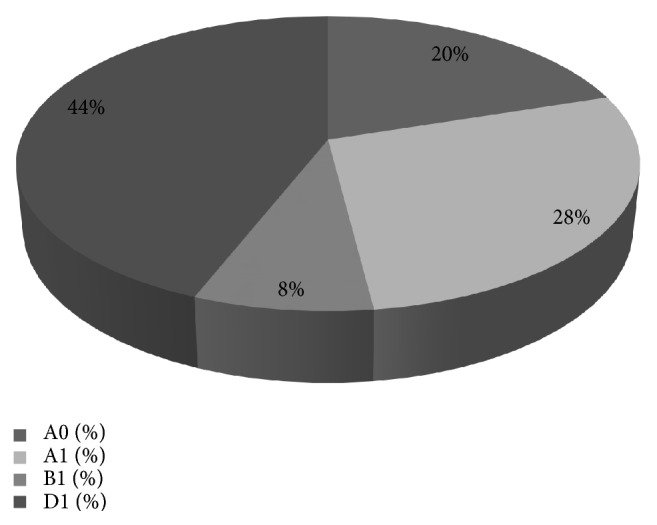
Percentage of strains by phylogenetic group.

As regards phylogenetic groups, D1 was the most prevalent (44%), followed by group A1 (28%), A0 (20%), and lastly B1 (8%); notably, no B2 group strains were observed (Figure 1).

In accordance with the absence of group B2 strains, ST131 was absent among our isolates, which all tested negative for the SNPs in the two genes of interest.

A limited number of virulence factors were observed, and these were concentrated in phylogenetic group D1. In fact, this group included all the strains with the* kpsMII* gene and the majority with* iutA* gene.* PapA* was found in one strain, belonging to group A1.

**Figure 2:**
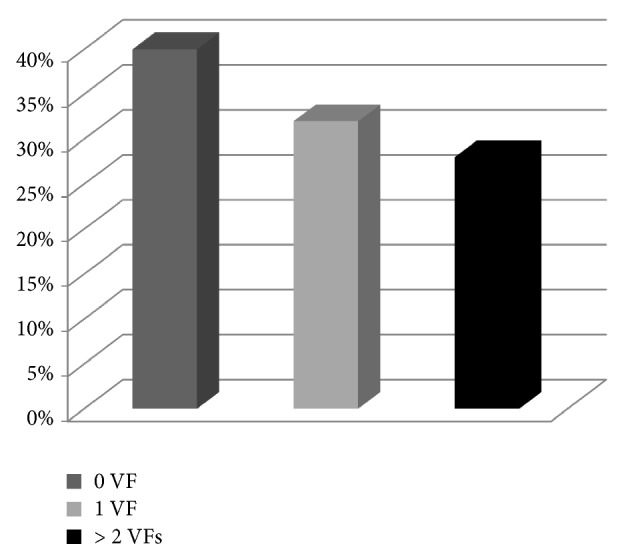
Percentages of strains possessing VFs.

Therefore, 7 of the 25 isolates (28%) met the inclusion criteria for ExPEC; that is, they had at least two virulence factors, according to Johnson et al. [18]. Eight isolates had one virulence determinant (Figure 2).

The genes* aac(6')-Ib-cr*,* qnrA*,* qnrB*, and* qnrS* encoding quinolone resistance were observed in only 20% of our isolates. In particular, both* qnrA* and* aac(6')- Ib-cr* genes appeared in 2 strains, while* qnrB *and* qnrS *were present in 1 strain each. 4 of these resistance determinants were found in group A and one in group D1; specifically, only one was found in pork meat (i.e.,* qnrB*), while the other determinants were found in strains originating from poultry.

Phenotypic resistance patterns are summarized in Table 1. Among the 25 isolated* E. coli* strains, notably 80% were found to be resistant to amoxicillin–clavulanic acid. A high prevalence was found for tetracyclin and sulfamethoxazole–trimethoprim also, both present in 72% of samples. Interestingly, none of the strains were observed to be resistant to the cephalosporins and carbapenem tested. The most common pattern was resistance to amoxicillin–clavulanic acid, tetracyclin, and sulfamethoxazole–trimethoprim, which was detected in 14 of the 25 strains (56%). Hence, 19 strains (76%) can be considered multidrug resistant, according to the criterion utilized [24].

**Figure 3:**
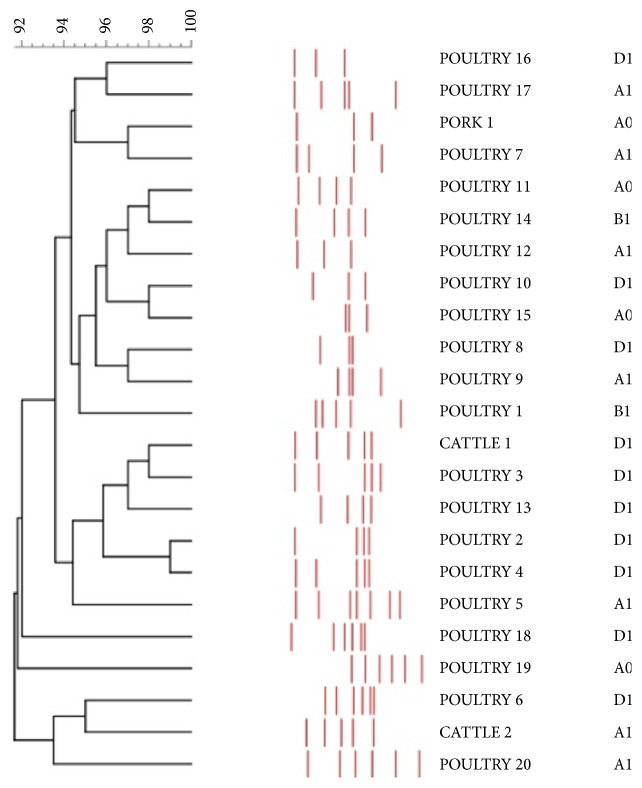
Dendrogram obtained by ERIC-PCR of strains.

Finally, ERIC PCR showed quite a high level of heterogeneity, except for two pairs of strains (2 pork strains and 2 poultry strains), which shared the same patterns of bands and hence are displayed only once (Figure 3).

## 4. Discussion

The steady increase in the prevalence of quinolone-resistant ExPEC isolates is particularly alarming due to their spread as opportunistic pathogens and suggests the need to deepen our knowledge of their source, reservoirs, and transmission pathways.

Poultry meat was highly contaminated with* E. coli* resistant to quinolones (91.3% of samples). The percentage of contaminated pork and beef samples was lower, in agreement with the literature, according to which not only does the poultry show the highest quinolone resistance in comparison to other types of meat but also the highest prevalence of MDR [25–27]. Lower resistance levels for beef, whose resistant strains accounted for 15.3% in this study, have also been observed in other investigations from different parts of Europe [28, 29].

The most common antibiotic classes used in bred chickens are penicillins, tetracyclines, sulfonamides and quinolones [6]. Accordingly, in our study, the highest resistance prevalence was found for amoxicillin-clavulanic acid, sulfamethoxazole–trimethoprim, and tetracycline. Carbapenems are restricted to human use, but they were investigated in this study, as required by 2013/652/EU [30], since small resistance spots are, albeit slowly, starting to emerge [31], above all in poultry, where a small percentage of carbapenemase-producing* E. coli* were detected from broilers and its meat in two European countries.

Although veterinary use of cephalosporins is permitted by law, notably our strains did not exhibit resistance to them. While the poultry industry in Italy renounced the use of III and IV generation cephalosporins in 2009, the other farming industries continue to use these antimicrobials for a rather wide range of diseases. Their consumption of this antibiotic class is one of the highest in Europe and has shown a slightly increasing trend since 2010 [32]. Our findings relating to cephalosporins reflect other data in the literature. Wasyl et al. [33] and Alvarez-Fernandez et al. [25] reported a very low incidence of resistance to these antibiotics (0-3.8%), and Pavlickova et al. [11] did not describe any strain as resistant to cefotaxime and cefuroxime. In contrast, another study from Sicily, but based on Italian meat, found a high prevalence of cephalosporin resistance in its strains [17].

We found a higher prevalence of phylogenetic group D1 strains (44%), followed by A1 (28%), A0 (20%), and lastly B1 (8%); these latter groups (A and B1) are usually associated with environmental and commensal strains in humans [34], while the phylogenetic groups B2 and to a lesser extent D are related to extra-intestinal pathogenic strains. Furthermore, in this study we found no evidence in the analyzed foods of an animal ST131 reservoir, in accordance with observations by other authors who only sporadically detected ST131 in farm animals [35, 36]. In fact, although other sources have been identified as ST131 vehicles, a greater prevalence of human compared to animal colonization has been observed [37].

Our isolates did not show a wide variety of VFs, as mainly the* iutA* receptor, present in 15 strains out of 25 (60%), and, to a lesser extent,* kpsMII*, present in 6 strains (24%), were found;* papA* gene was found in just 1 isolate. The presence of virulence genes in these strains is worrisome because it suggests a high probability of pathogenicity, according to Johnson et al.'s [18] ExPEC definition. VFs, therefore, greatly increase the health threat these strains already pose as carriers of antibiotic-resistance genes through the food chain. Specifically, 28% of isolates possessed two virulence factors (i.e., ExPEC). Lyhs et al. [38] classified 22% of strains as ExPEC in a study focused on poultry meat sold at retail stores, while Xia et al. [39] observed an even lower percentage (20.2%) in the same sample type.

However, Johnson et al.'s above-mentioned classification for determining the pathogenicity of microorganisms may not be exhaustive, as there may be other unexamined factors conferring pathogenic potential. For instance, in a study by Fasciana et al. [40], many UPEC strains, all isolated from pathological urine samples, did not exhibit any of the VFs indicated by Johnson et al. [18]. Hence it is highly likely that the number of ExPECs among farm animals has been underestimated.

As regards antibiotic resistance, since all the isolates in question exhibited phenotypic resistance to quinolones, other resistance mechanisms may explain the rather low prevalence of the PMQR genes investigated (20%). Indeed, as the most common mechanisms in animal isolates are chromosomal mutations in type II topoisomerase (*parC* and/or* gyrA* genes) [41–43], or in regulatory proteins (e.g., MarA, SoxRS, and Rob) associated with upregulation of efflux pumps, such as AcrAB-TolC, and downregulation of porin, reducing quinolone influx [44], we assume that these mechanisms were responsible for resistance in our study as well. In addition, other potentially involved resistance determinants, though less frequent, are due to plasmid-encoded qepA and oqxAB membrane transporters [45, 46].

Genotyping by ERIC-PCR revealed 23 banding profiles; these results support a high genetic heterogeneity, which is an alarming fact, showing a multiple onset of MDR strains despite the restricted area of sampling.

This study, although numerically limited, emphasizes the already clear need to improve strategies to prevent the spread of antibiotic resistance and to reduce the amount of antibiotics used.

The high prevalence of resistant strains in this study, despite not all of them being classified as ExPEC, poses a direct risk, as these strains can subclinically colonize the consumer's intestinal tract until advantageous circumstances favour an extraintestinal infection, or an indirect risk, potentially contributing resistance genes to human indigenous microbiota [47].

For instance, a subset of human ExPEC strains, isolated by Fasciana et al. [40] from UTIs in Palermo, turned out to be non-ST-131 (33%) and were not resistant to cephalosporins (32.4%). Furthermore, the most prevalent VFs observed included* KpsMII* (32%) and* iutA* (83.8%), which were reported to be more common in non-ST131 strains. These data are limited and do not allow epidemiological considerations, but they do underline, albeit partially, that it is not possible to exclude a zoonotic origin for at least a small subset of human ExPEC infections.

Given the considerable public health threat that ExPEC represents, further long-term investigations are needed to give us more insight into the epidemiologic relationship between human and food-origin* E. coli,* and to clarify capacity for interspecies transfer.

With regard to animal production systems, a review of farm management is essential, especially as far as intensive farming is concerned, combining good practices and applying good hygiene measures and animal welfare in order to reduce the use of antimicrobials (i.e., an efficient antimicrobial stewardship), thus acting on reservoirs of antibiotic resistance. At present, intensive farming systems rely on a routine use of antibiotics, creating reservoirs of antimicrobial resistance genes that could spread in the environment or to different hosts. In fact, antibiotics are often used as prophylactic prevention measures, for mass treatment that is not associated with a specific diagnosis or for preventable diseases, in a way that is no longer sustainable. In order to deal with this antimicrobial resistance emergency, different levels of safety measures must be considered, including “tertiary prevention” (i.e., increasing the ability of the animals' immune system to respond to infections) [48] and vaccines formed on widely conserved antigens [49, 50].

In addition, according to the farm-to-fork concept, it is important that also slaughterhouses and food handling practices are taken into account in the attempt to reduce foodborne transmission. Hence, an integrated implementation of GMP (Good Manufacturing Practices) and GHP (Good Hygienic Practices) should be applied throughout the production, processing, and consumption stages, and consumer awareness should be raised.

## Figures and Tables

**Table 1:** Resistance patterns observed in all the strains.

Resistance Pattern	N. isolates (%)
CIP, NOR, LVX (Fluoroquinolones only)	3 (12%)
CIP, NOR, LVX, AUG	3 (12%)
CIP, NOR, LVX, AUG, TE	1 (4%)
CIP, NOR, LVX, AUG, SXT	1 (4%)
CIP, NOR, LVX, AUG, SXT, TE	16 (64%)
CIP, NOR, LVX, AUG, SXT, TE, CN	1 (4%)

## Data Availability

The data used to support the findings of this study are available from the corresponding author upon request.
